# Towards malaria control and elimination in Ghana: challenges and decision making tools to guide planning

**DOI:** 10.1080/16549716.2017.1381471

**Published:** 2017-10-16

**Authors:** Timothy Awine, Keziah Malm, Constance Bart-Plange, Sheetal P. Silal

**Affiliations:** ^a^ Modelling and Simulation Hub, Africa, Department of Statistical Sciences, University of Cape Town, Cape Town, South Africa; ^b^ South African Department of Science and Technology/National Research Foundation Centre of Excellence in Epidemiological Modelling and Analysis (SACEMA), University of Stellenbosch, Stellenbosch, South Africa; ^c^ National Malaria Control Program, Ministry of Health, Accra, Ghana; ^d^ Tropical Disease Modelling, Nuffield Department of Medicine, University of Oxford, Oxford, UK

**Keywords:** Malaria, mathematical models, interventions, policy, planning, control

## Abstract

Ghana is classified as being in the malaria control phase, according to the global malaria elimination program. With many years of policy development and control interventions, malaria specific mortality among children less than 5 years old has declined from 14.4% in 2000 to 0.6% in 2012. However, the same level of success has not been achieved with malaria morbidity. The recently adopted 2015–2020 Ghana strategic action plan aims to reduce the burden of malaria by 75.0%. Planning and policy development has always been guided by evidence from field studies, and mathematical models that are able to investigate malaria transmission dynamics have not played a significant role in supporting policy development. The objectives of this study are to describe the malaria situation in Ghana and give a brief account of how mathematical modelling techniques could support a more informed malaria control effort in the Ghanaian context. A review is carried out of some mathematical models investigating the dynamics of malaria transmission in sub-Saharan African countries, including Ghana. The applications of these models are then discussed, considering the gaps that still remain in Ghana for which further mathematical model development could be supportive. Because of the collaborative approach adopted in their development, some model examples Ghana could benefit from are also discussed. Collaboration between malaria control experts and modellers will allow for more appropriate mathematical models to be developed. Packaging these models with user-friendly interfaces and making them available at various levels of malaria control management could help provide the decision making tools needed for planning and a platform for monitoring and evaluation of interventions in Ghana.

## Background

Globally, about 3.2 billion people were at risk of malaria infection in 2015, with the number of cases being 214 million. Deaths attributable to malaria were estimated to be 438,000, most of which occurred in sub-Saharan Africa.[1] According to the global malaria elimination program classification, Ghana and much of West Africa are currently classified among nations considered to be in the control phase.[2] Since the call for malaria elimination was made about a decade ago, Ghana and other sub-Saharan African countries have made great strides to achieve reductions in the burden of diseases associated with malaria.[3] Development and implementation of successive strategic plans of action, intervention policies and a boost in financial support over the years in Ghana have led to some reduction in the burden of malaria.[4]

The recently adopted 2015–2020 Ghana Malaria Strategic Plan aims to reduce malaria burden by 75.0%.[4,5] However, readily available mathematical models to support decisions for planning and subsequent evaluation of these strategies remains a challenge, given the limited research undertaken in this field in Ghana. This and other gaps identified by the National Malaria Control Program (NMCP), such as the lack of evidence for rational deployment of interventions, evaluation of their impact and cost effectiveness of these interventions, makes it important to further explore the usefulness of mathematical models to help support malaria control efforts.[5]

Historically, mathematical modelling has provided a platform for generating the evidence needed for planning and decision making.[6] If such models are developed in collaboration with the National Malaria Control, Ministry of Health and Ghana Health Service, they could guide the optimal use of various interventions alone or in combination, in various transmission settings. In particular, their application may include evaluation of coverage levels that are needed to achieve set targets. They may also be used to evaluate impact and cost effectiveness of intervention packages, to guide decision making with regards to e.g. scaling down once the desired levels of disease burden have been attained.[6,7]

This paper will describe the malaria situation in Ghana and give a brief account of how mathematical models can be used in the Ghanaian context towards a more informed malaria control and elimination programme.

## A brief malaria profile of Ghana

Malaria remains a serious health challenge in most of sub-Saharan Africa.[8]

**Figure 1.**
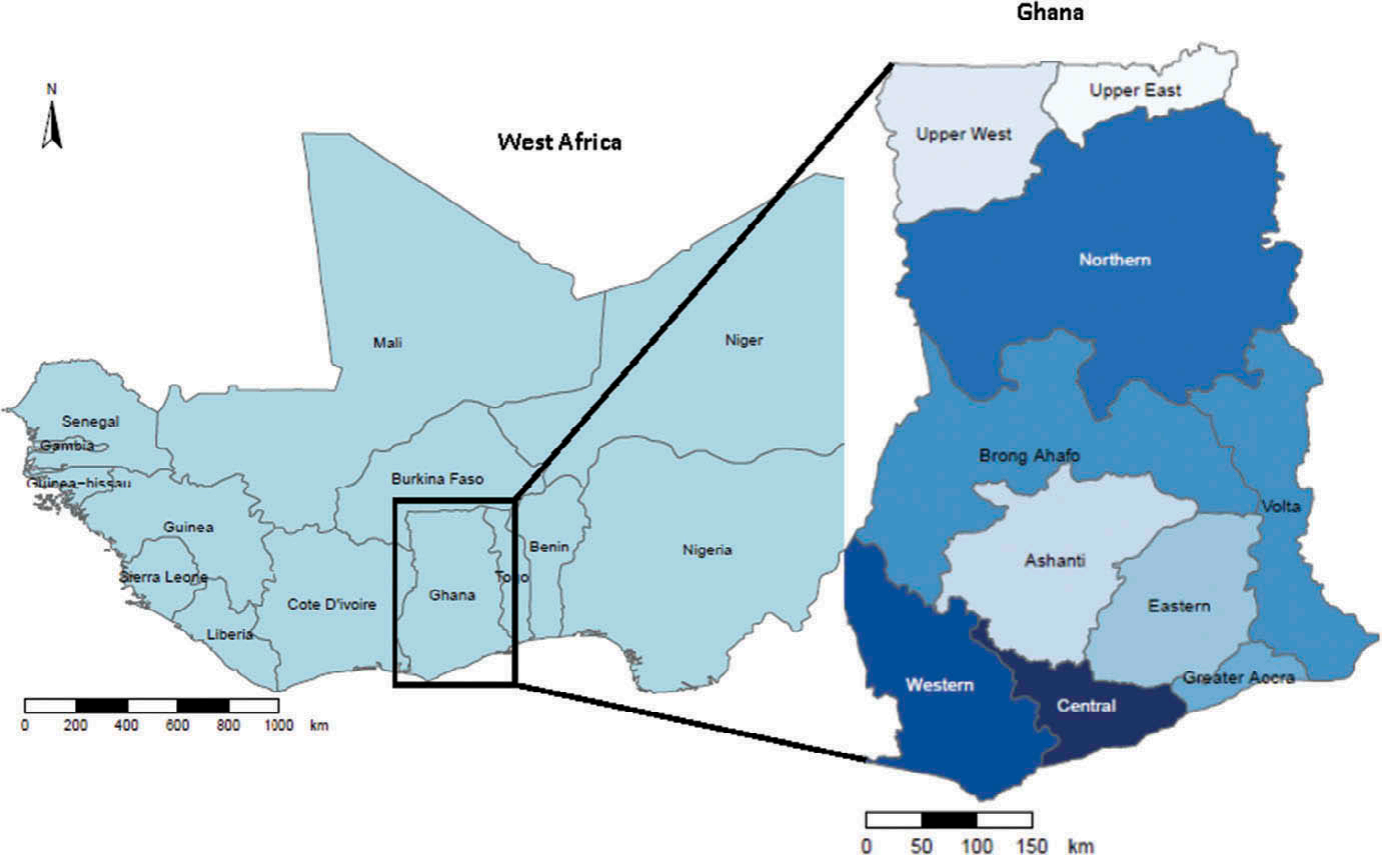
Map of West African sub-region showing the location of Ghana (shapefiles from MapCruzin.com).

The West African sub-region, of which Ghana is a part (Figure 1), is estimated to have more than 300 million people at risk of malaria infection. Thus, the sub-region accounts for about half of the global burden.[8]

Malaria is endemic in Ghana, which means that all 27 million inhabitants [1] are susceptible to malaria infection.[5] The incidence of malaria still accounts for 40.0% of all outpatient attendance,[9] with the most vulnerable groups being children under 5 years of age and pregnant women.[4,5] Parasite prevalence among children decreased marginally from 47.0% to 45.0% from the 2000 to 2010.[5] Nationwide surveys in 2011 and 2014 also estimated population average parasite prevalence to be 28.0 and 27.0% respectively among children 6–59 months of age.[10,11] Furthermore, malaria attributable mortality among this age group reduced from 14.4% in 2000 to 0.6% in 2012.[4]

Within Ghana malaria transmission is heterogeneous and differs along varying ecological zones. Parasite prevalence is highly seasonal, peaking in a single wet season (June–October) in the northern savannah area.[12–16] However, in both forest and coastal ecological areas, malaria parasite prevalence peaks twice in a year.[12,13,17]

Though a variety of vector species have been identified in Ghana, *Anopheles gambiae* and *Anopheles funestus* are the most dominant, while *Plasmodium falciparum* constitutes more than 95.0% of the parasite species. Low-level infections of *Plasmodium malariae* and *Plasmodium ovale*, mostly occurring as mixed infections with *P. falciparum*, have been observed, but *Plasmodium vivax* is yet to be reported.[1,5]

**Figure 2.**
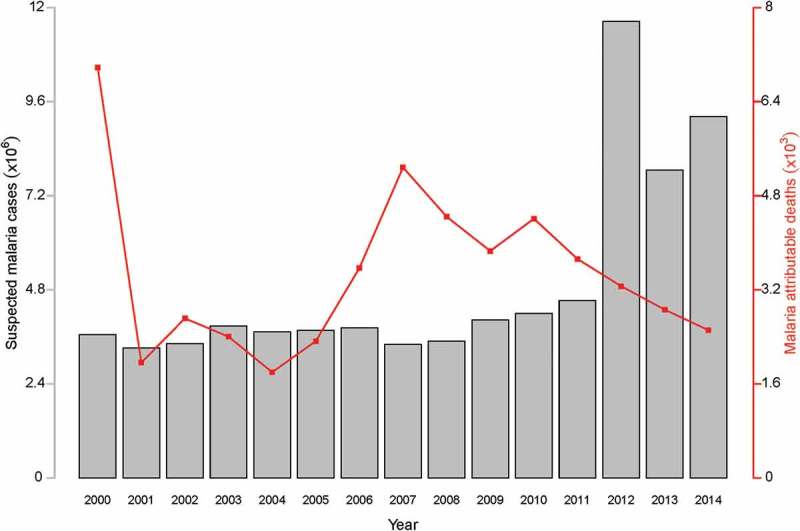
Malaria situation in Ghana: graphs of suspected malaria cases and malaria attributable deaths (data source: World Malaria Report 2015).

The profile of annually suspected cases reported and deaths attributable to malaria at various health facilities in Ghana is shown in Figure 2.

## Brief history of malaria interventions

Ghana has a rich history of malaria control interventions dating back to the pre-independence era. These interventions mainly targeted either the parasite in the host (human beings) or the vector (anopheles mosquitoes).[5]

Interventions in the form of medications targeting the parasite in the human host such as amodiaquine-pyrimethamine, daraprim or pyrimethamine, primaquine, lapudrine and chloroquine were tested and administered in various ways before independence in the 1950s.[5]

In the years between the 1950s and 2000, the use of monotherapies including chloroquine and other medications such as quinine were widespread. As in many endemic countries, the use of chloroquine and other interventions persisted until widespread parasite resistance to chloroquine was reported. This necessitated a policy recommendation for a change.[18] Thus a treatment policy change was made in 2004 to replace the first-line treatment regime to the artemisinin combination therapies (ACTs), i.e. artesunate-amodiaquine, for treatment of uncomplicated malaria. This treatment policy has since been revised twice, in 2007 and 2009, to include other ACTs, namely artemeter-lumefantrine and dihydroartemisinin-piperaquine.[19]

Vector control activities have also been in use since the pre-independence era. Interventions such as indoor residual spraying (IRS) and bed nets have been used before but not on a wide scale.[4] These interventions were predominantly deployed in major cities. Insecticide Treated Bed nets (ITNs) were deployed nationally in 2004, following evidence from field trials of their effectiveness in 1996 in Ghana and elsewhere. A policy was also made to subsidise delivery of ITNs in 2007.[5]

**Figure 3.**
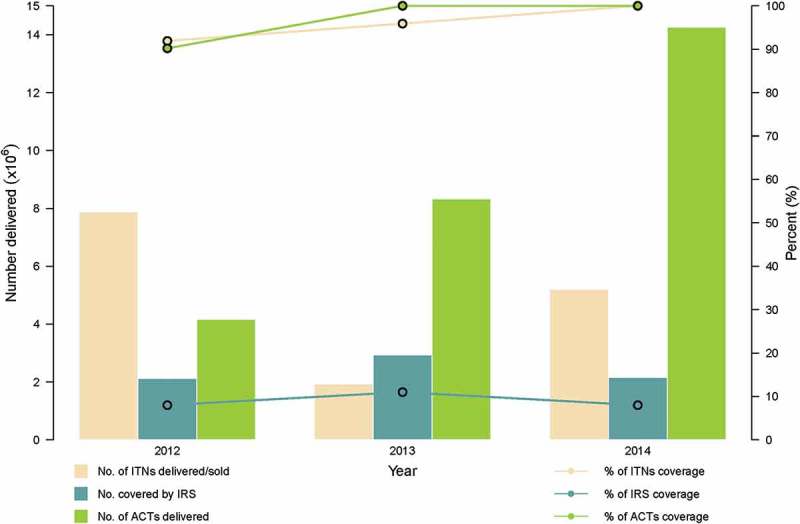
Malaria interventions delivery and coverage from 2012 to 2014 (data source: World Malaria Report 2015).

Since 2005, IRS activities have been recommended; however their deployment has been on a limited scale.[1] Figure 3 shows the distribution and coverage levels of ITNs, IRS and ACTs from 2012 to 2014. The coverage levels for ACTs and ITNs have gradually risen to 100%.

Despite the high levels of coverage of the interventions, the level of malaria morbidity in Ghana remains relatively high,[9] making it and other West African states unlikely to eliminate local transmission of the disease before 2030.[2] Ghana has adopted a strategic action plan with a 2020 target to, among other things, reduce the burden of malaria in the population by 75.0%.[4] This requires multidisciplinary research if the ultimate goal of elimination by 2030 is to be attained.

Within the frame of the 2030 targets set by the Global Malaria Programme (GMP), appropriate mathematical models could help to find deployment strategies for existing and new intervention packages across the three distinct epidemiological zones of the country. These models should also provide a suitable platform for monitoring and evaluating the impact of deployed interventions and track progress towards set goals at both the zonal and national level.

## Mathematical modelling of malaria transmission dynamics

Mathematical modelling techniques have been in use for centuries to study transmission dynamics of various infectious diseases.[20]

The foundations of modern mathematical modelling techniques specifically for malaria transmission dynamics were laid by Ronald Ross in the early part of the eighteenth century when he demonstrated the link between mosquitoes and malaria transmission and subsequently developed a simple mathematical model to describe transmission of malaria.[21,22] The Ross models were followed by more complex models that have helped in furthering understanding of malaria transmission, and have guided policy development for disease control in different settings.[23,24]

In Ghana, attempts have been made to develop similar mathematical models to describe the dynamics of malaria. Using ordinary differential equations, compartments representing susceptible, exposed, infected and recovered/removed (SEIR) and susceptible, exposed infected (SEI) for both human and mosquito populations respectively were developed. The focus of these models was on investigating the basic reproductive number, conducting stability analyses and simulation studies to determine when Ghana could achieve malaria free status.[25] The second model, with same structure, sought to test the impact of interventions on malaria transmission in Ghana.[26] These two models were developed and parameterised using country-wide annually aggregated health facility reported malaria incidence data.

Another model was developed with two infectious classes for the human population using differential equations.[27] This model was used to derive the conditions for disease free and endemic equilibria and compared model results with those developed by Ross,[22] Chitnis et al. [28] and Ngwa et. al.[29]

Modifying the Kermack–Makendrick model,[30] a susceptible, infected, recovered/removed susceptible (SIRS) model was used to describe the transmission dynamics of malaria in a municipality in the forest region of Ghana.[31] Municipal-level health facility reported malaria cases were used to fit the model in 2014, which involved investigating the prevalence of malaria and also determining the disease-free equilibrium state for the Techiman municipality.[31]

While the results of all the aforementioned models were informative, potentially key factors of malaria epidemiology in Ghana are yet to be considered. Factors including the varying epidemiological settings of populations across the country and available monthly aggregated data from all health facilities in all districts rather than the annualised data could offer more insights to the transmission dynamics of malaria in Ghana at the specific ecological zone level, given the varying meteorological factors across the country that lead to different incidence of malaria.[12]

Despite the potential usefulness of country-specific mathematical models in studying infectious diseases such as malaria, there seems to be no evidence to suggest that they have guided malaria intervention strategies in Ghana.

Models have been developed elsewhere in Africa where the diversity in malaria transmission across regions is taken into account through the incorporation of meteorological factors such as rainfall and temperature. These models were successfully used to investigate the impact of interventions such as IRS and ITNs on the transmission of malaria.[32–35]

The earliest mathematical model for malaria transmission in Africa was the Garki model, which was developed and tested in a large field trial in northern Nigeria in 1974.[36] The Garki model investigated the relationship between entomological variables, prevalence and incidence of malaria in the population and how these vary by season and age. The benefits of deploying interventions singly and in combination in the region were also explored using this model. Results of the Garki model provided evidence for future malaria control strategies, especially for the sub-Saharan African region of Africa.[37]

Other models in Nigeria, Ivory Coast and Mali aided the investigation of malaria transmission dynamics,[38] the conduct of stability analyses,[39,40] investigation of the impact of ITNs and malaria acquired immunity [41] and the study of the relationship between temperature and precipitation with malaria incidence.[42]

Mathematical models have also been successfully developed in eastern Africa. Some investigated malaria transmission dynamics in mosquitoes and humans,[43–45] others were used to evaluate the impact of interventions against the disease [43,46] and other models tested the adequacy of age structure in explaining transmission of disease and the impact of varying transmission settings.[35,43,47] The effect of migration on the transmission of malaria have also been modelled in Kenya.[43,48]

Even though South Africa is largely malaria free, the dynamics of malaria in the northern provinces of South Africa were investigated using various mathematical models.[49–51] These models also investigated the impact of various malaria interventions, the effect of cross border migration (between Mozambique and South Africa) on the persistence of malaria in South Africa and prospects of elimination.

While the results from these studies are useful, one major drawback is often the seeming lack of awareness of these models by programme managers at the national and district level. Providing these models, built with the involvement of programme managers, on user friendly platforms will be the next necessary step that may prove to be invaluable in helping to combat incidence of malaria on the African continent.

## Rationale

The National Malaria Control Program (NMCP) in Ghana has made laudable strategic plans to reduce the burden of the disease by 75% across the country by 2020. The aim is to achieve this goal by intensifying the distribution of treated bednets (ITNs) and scaling up monitoring and evaluation (M&E) activities.[4]

However, the NMCP will need the tools to adequately justify the approach in which interventions are deployed across different epidemiological settings across the country.[5] Their inability to effectively assess the impact of interventions with least financial commitment, other than conducting field studies that tend to be more expensive has been highlighted. [4]

Regardless of the availability of health facility level reported cases on the DHIMS, which are analysed periodically to assess progress of malaria morbidity at the regional level, these analyses can only be carried out retrospectively. The ability to provide evidence prospectively to support decision making that could form a basis for intervention deployment may not be available with the usual approach of analysing the data.

The retrospective limitations of conventional approaches may also not allow evaluation of the impact and cost effectiveness of existing or new interventions [5] without gathering data in expensive field studies.

Although a few attempts have been made to develop malaria transmission models in Ghana, there are some limitations in their implementation. While some models [26] aim at Ghana in general, they do not consider the varying epidemiological settings accounting for transmission variability due to local factors such as climatic and environmental factors. Other models [25,27,31,52–54] were developed on a smaller scale and more tailored to data from specific districts. Almost all the models developed in Ghana, as mentioned above, used data from communities located in either the forest or coastal ecological zones. None considered the transmission dynamics of the savannah zone of northern Ghana where the transmission of malaria is relatively more intense.[10]

This project therefore aims to develop a suite of mathematical models that could be used to predict the transmission of the disease in all the different epidemiological/ecological zones of Ghana and can also be used to assess the optimal impact of interventions individually or in combination. The study also aims to extend these models for evaluation of the cost effectiveness of interventions. Finally a friendly user interface visualising the data and incorporating these models will be developed for use at various levels of malaria control. These tools will support the development of relevant policies for the effective control of malaria with limited resources.

## Hypothesis

A spatially explicit population level dynamic mathematical model that takes into account the varying epidemiology of malaria morbidity in Ghana, validated using routine health facility data from all districts, will be developed to investigate the impact of malaria interventions and support planning.

## Aim and objectives

To develop and validate a suite of mathematical models that can be used to predict malaria transmission and to investigate the impact and cost of malaria interventions in Ghana.

Specific objectives:

- Determine the relationship between malaria morbidity and weather variables in Ghana using statistical methods.
- Develop dynamic spatially explicit population level mathematical models and assess the optimal combination of various interventions in different epidemiological settings in Ghana.
- Evaluate the cost effectiveness of these interventions on various populations.
- Assess prospects of achieving proposed local and international set targets, based on the formulated mathematical models.
- Develop an interactive and user friendly interface of the mathematical models using the R software and making them available to policy makers and managers of malaria control, at the regional and national level.

## Methods

The proposed mathematical models will be developed using nonlinear ordinary differential equations. The basic model structure will include coupling compartments for populations that are susceptible, infected and recovered for humans and susceptible, infected and infectious for mosquitos. Due to the endemicity of malaria transmission in Ghana,[5] the concepts of immunity and superinfection will be factored into the model design.[55]

Transitions between compartments will be modelled incorporating appropriate rate parameters estimated from observed data or from the literature. Seasonality of malaria transmission will be captured through forcing functions or functions of meteorological variables of the various ecological zones as covariates.

The impact of interventions will be tested by simulating various levels of intervention coverages (singly or in combination) on the potential reduction on transmission of disease in the various zones. Similarly the prospects of reducing the prevalence and incidence of malaria to desired set targets, by the NMCP and those for malaria elimination, will be tested based on the impact of varying coverage levels of interventions on transmission.

Cost effectiveness of various interventions will also be calculated based on the net intervention cost. That is, benefits in terms of cost reduction derived to the health system following implementation of the interventions. Average cost effectiveness ratio (ACER) will then be calculated as the ratio of the net cost of interventions and the net effect of interventions.[56] Other desired outcomes with policy implications including cost per case, cost per case averted, cost per death averted and disability adjusted life years (DALYs) will also be calculated.

Models will be fitted with the aim to matching the features and trajectory of observed monthly reported cases within each zone and the best parameter set obtained through maximum likelihood methods.

## Data

Clinical data for this study will be obtained from the health facility records of confirmed uncomplicated and severe malaria cases, malaria attributable deaths and records of pregnant women confirmed to have malaria. These data are captured and stored on the DHIMS platform and are available to the NMCP. Aggregated monthly records of all confirmed cases of malaria for all age groups and districts from 2000 to 2016 will be used.

Data for the cost effectiveness analysis will also be obtained from NMCP and other published literature. These will include budget estimates and previous expenditures for malaria interventions and malaria specific activities and programs in Ghana.

For the purposes of studying the relationships between malaria morbidity and meteorological variables, monthly average precipitation (mm) and temperature (minimum and maximum) (°C) from all 10 regions in Ghana for the period 2000–2016 will be used. These data will be obtained from the Ghana Meteorological Agency (GMET).

## Ethical considerations

Ethical approval will be sought from the Navrongo Health Research Centre Institutional Review Board and the Faculty of Science Ethics Committee of the University of Cape Town. Permission to use the data will also be sought from the NMCP.

## Expected outcomes

Expected outcomes will include the following proposed articles that will be submitted for peer review and publication:

1. Towards malaria control and elimination in Ghana: challenges and decision making tools to guide planning.
2. Investigating the relationship between seasonal dynamics of reported malaria cases and weather variability in Ghana.
3. Accounting for regional transmission variability and the impact of malaria interventions in Ghana (population level mathematical approach).
4. Cost effectiveness of malaria control interventions in Ghana: a mathematical modelling approach.

## Discussion

There seems to be no evidence of country-specific mathematical models playing a role in supporting policy decision making with regards to malaria intervention strategy development, although a number of studies, as mentioned earlier, have been conducted as part of efforts to understand the epidemiology of malaria in Ghana.

One of reasons accounting for this may include the non-availability of data, prior to the DHIMS platform, needed to be used to validate model parameters. Another reason could be limited interaction between modellers and policy makers. Tapping the full potential of mathematical models to support policy may require a collaborative effort between model builders and malaria control stakeholders such as the National Malaria Control Program, Ministry of Health, and Ghana Health Service.

Examples of collaborative research into building mathematical models include one in Cambodia and another in Thailand. In Cambodia, the National Institute for Health designed and implemented, collaboratively with policy makers and other stakeholders of malaria control, a malaria early warning system supported by seasonal climate forecasting, weather monitoring, statistical and dynamic models. The system was implemented at the municipal level and was made available for use by malaria control managers.[57] A user-friendly web-based mathematical model was also developed by the Malaria Elimination Team from Mahidol-Oxford Tropical Medicine Research Unit at the University of Mahidol in Thailand. Their model platform made allowance for policy makers and malaria control managers to examine the elimination potential of various malaria control interventions, alone and in combination, in a variety of malaria transmission settings. It was designed to allow users input their data, run the models over the internet and obtain various outputs for different parameter settings.[58]

In Ghana, developing a mathematical model to support policy development will require detailed parameterisation and validation using results of the numerous epidemiological studies available in published literature as well as data unpublished and available to malaria control stakeholders. The recent analyses undertaken by the NMCP to generate an updated malaria prevalence map for Ghana using data from 1960 to 2011 is one very useful source of data. Additionally, aggregated monthly health facility data maintained on the DHIMS [5,59] will support model fitting and parameter validation. Data for other risk factors such as monthly rainfall and temperature, socio-economic classification of the population (rural or urban), demographic and migration data will also be useful for model parameterisation.

Therefore, the mathematical model development process proposed in this study will consider both population and sub-population level dynamics of malaria transmission along the varying epidemiological settings of Ghana. This will be done by factoring in local disease transmission dynamics of malaria and also by engaging policy makers, such as the NMCP to gain more insights, including the practical difficulties of intervening in malaria transmission in the country.

## Conclusion

It is envisaged that the process of conducting this research collaboratively with the NMCP in Ghana will afford the opportunity to support policy makers and stakeholders in the field of malaria control through country-specific mathematical models. These models should be useful for investigating malaria transmission dynamics for purposes of disease control and policy evaluation.

Specifically, the challenges that may be addressed by the earlier proposed modelling venture are well articulated by the NMCP of Ghana in the following statement: ‘Among the challenges facing the future of effective control include a more rational basis for stratified intervention delivery, better planning information and an ability to generate sufficient evidence to demonstrate impact and value for money’ (p. 1) .[5]

More importantly, the opportunity for interaction between malaria control experts and modellers will create a platform for information sharing, presenting a unique platform for the development of more practically focused models for guiding malaria control activities in Ghana.

Subsequently, packaging these mathematical modelling tools into user friendly interfaces and making them available for use by malaria control management teams at various levels across Ghana will be the way to exploit synergies for a common goal of a possible malaria elimination by 2030 as envisaged by the Global Malaria Programme.

## Paper context

This paper highlights some of the challenges facing the NMCP in its effort to controlling the incidence of malaria and identifies and discusses how some support could be harnessed, collaboratively between modellers and managers of disease control in Ghana, using mathematical modelling techniques. The approaches discussed in this paper may be found useful to Ghana and also to other sister West African nations.
